# Draft Genome Sequence of Lactiplantibacillus plantarum DMR09, Isolated from Spontaneously Fermented *Dahi*

**DOI:** 10.1128/mra.01097-22

**Published:** 2023-05-30

**Authors:** Ranjan Kaushal Tirwa, Anil Bhattarai, Buddhiman Tamang

**Affiliations:** a Department of Microbiology, Nar Bahadur Bhandari Degree College, Tadong, Sikkim, India; b Department of Medical Biotechnology, Sikkim Manipal Institute of Medical Sciences, Sikkim Manipal University, Tadong, Sikkim, India; c Department of Microbiology, School of Life Sciences, Sikkim University, Tadong, Sikkim, India; University of Southern California

## Abstract

The draft genome sequence of Lactiplantibacillus plantarum DMR09, which was isolated from spontaneously fermented homemade cow *dahi*, was analyzed. The genome profile of L. plantarum DMR09 revealed 3,315,630 bp and a GC content of 44.5%, with 3,036 protein-encoding genes, 72 rRNA genes, and 65 tRNA genes.

## ANNOUNCEMENT

Lactiplantibacillus plantarum is a lactic acid-producing Gram-positive bacterium that is known for its ecological and metabolic plasticity, which makes it an ideal candidate for use in the pharmaceutical, dairy, food, and beverage industries (1, 2). A useful strategy for assessing its probiotic potential involves whole-genome sequencing (WGS)-based investigations (3).

In this study, L. plantarum DMR09 was isolated from spontaneously fermented homemade cow dahi from the Himalayan region of India, as described by Tirwa et al. (4). The genomic DNA of DMR09 was extracted after growing the culture in MRS medium at 37°C, using the phenol-chloroform method (5). Sequencing of the 16S rRNA gene was performed using 27F (5′-AGAGTTTGATCMTGGCTCAG-3′) and 1492R (5′-TACGGYTACCTTGTTACGACTT-3′) primers, with the PCR conditions described by Chagnaud et al. (6). The NCBI BLAST (7) results for the 16S rRNA gene sequence showed 99.8% similarity with L. plantarum JCM 1149 (GenBank accession number NR_115605.1). The genomic DNA of DMR09 was extracted using the QIAamp DNA minikit (Qiagen) following the manufacturer’s instructions. The DNA library was prepared with the NEBNext Ultra DNA library preparation kit (New England Biolabs), and the paired-end sequencing was performed using the Illumina HiSeq X Ten platform (2 × 150-bp reads). FastQC v0.11.9 was used to identify and remove low-quality sequence reads, which ultimately produced 15,779,818 reads, with a mean length of 134 bp (8). *De novo* genome assembly was performed using Unicycler v0.4.8 (9). The assembly statistics were obtained with QUAST v4.0 (10). The tRNA genes were identified using ARAGORN v1.2.38 (11) and tRNAscan-SE v2.0 (12). PlasmidFinder v2.0 (13) was used to check noncore genomic elements. The contigs were further processed for genome annotation using NCBI Prokaryotic Genome Annotation Pipeline (PGAP) (14). To identify single-nucleotide polymorphisms (SNPs) within DMR09, the paired-end sequences were aligned with the reference genome of L. plantarum WCFS1 using BWA v0.7.12 (15). The aligned reads were sorted using SortSam of the Picard toolkit v3.0.0 (https://broadinstitute.github.io/picard). Read duplicates were removed using the Picard MarkDuplicates. SNPs and short indels were analyzed using SAMtools v1.16.1 (16). The output from SAMtools mpileup was piped to BCFtools v1.16 to call genomic variants. The BCFtools filter function was used to filter low-quality SNPs based on alternate allele strand bias (bias of >90%), quality (quality scores of <20), and depth (coverage depth of <10×). Default parameters were used for all software unless otherwise specified.

The draft genome sequence of L. plantarum DMR09 contained 3,315,630 bp in 58 contigs, with an *N*_50_ value of 231,289 bp and genome coverage of 765-fold. In total, 3,036 protein-coding genes, 72 rRNA genes, and 65 tRNA genes were predicted. The genome sequence of DMR09 contains 20,447 SNPs and 79 indels, with a GC content of 44.5%.

### Data availability.

The rRNA gene and the whole-genome shotgun sequences of DMR09 were submitted to NCBI GenBank with accession numbers MH588224.1 and JADANI000000000 (version JADANI010000000), respectively.
